# Expression and metabolism profiles of CVT associated with inflammatory responses and oxygen carrier ability in the brain

**DOI:** 10.1111/cns.14494

**Published:** 2023-10-30

**Authors:** Ling Kui, Yinming Jiao, Huimin Jiang, Guoyun Wang, Zongyu Li, Xunming Ji, Chen Zhou

**Affiliations:** ^1^ Shenzhen Qianhai Shekou Free Trade Zone Hospital Shenzhen China; ^2^ Laboratory of Brain Disorders, Ministry of Science and Technology, Collaborative Innovation Center for Brain Disorders, Beijing Institute of Brain Disorders, Beijing Advanced Innovation Center for Big Data‐based Precision Medicine Capital Medical University Beijing China; ^3^ Dehong People's Hospital Mangshi China; ^4^ Department of Neurosurgery, Xuanwu Hospital Capital Medical University Beijing China

**Keywords:** cerebral venous sinus thrombosis, heterogeneity, inflammation, ischemic strokes, metabolism, middle cerebral artery occlusion, oxygen, RNA‐seq

## Abstract

**Aim:**

As the main type of stroke, the incidence of cerebral venous thrombosis (CVT) has been rising. However, the comprehensive mechanisms behind it remain unclear. Thus, the multi‐omics study is required to investigate the mechanism after CVT and elucidate the characteristic pathology of venous stroke and arterial stroke.

**Methods:**

Adult rats were subjected to CVT and MCAO models. Whole‐transcriptome sequencing (RNA‐seq) and untargeted metabolomics analysis were performed to construct the transcriptome and metabolism profiles of rat brains after CVT and also MCAO. The difference analysis, functional annotation, and enrichment analysis were also performed.

**Results:**

Through RNA‐seq analysis, differentially expressed genes (DEGs) were screened. 174 CVT specific genes including *Il1a*, *Ccl9*, *Cxxl6*, *Tnfrsf14*, etc., were detected. The hemoglobin genes, including both *Hba* and *Hbb*, were significantly downregulated after CVT, compared both to the MCAO and Sham groups. Metabolism analysis showed that CVT had higher heterogeneity of metabolism compared to MCAO. Metabolites including N‐stearoyltyrosine, 5‐methoxy‐3‐indoleaceate, Afegostat, pipecolic acid, etc. were specially regulated in CVT. Through the immune infiltration analysis, it was found that CVT had a higher immune response, with the abundance of certain types of immune cells increased, especially T helper cells. It was important to find the prevalence of the activation of inflammatory chemokine, cytokine, NOD‐like pathway, and neutrophil extracellular trap.

**Conclusion:**

We explored and analyzed the gene expression and metabolomic characteristics of CVT, revealed the specific inflammatory reaction mechanism of CVT and found the markers in transcriptome and metabolism levels. It points out the direction for CVT early diagnosis and treatment.

## Graphical abstract

Adult rats were subjected to CVT and MCAO models. Multi‐omics (RNA‐seq and untargeted metabolomics) analysis were performed to construct the transcriptome and metabolism profiles of rat brains after CVT and also MCAO. The differential gene analysis, functional annotation, and enrichment analysis were also performed.

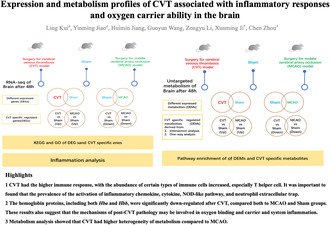

## 1 INTRODUCTION

Stroke is one of the leading causes of death and severe disability worldwide, can be classified as arterial or venous, according to the vascularity of the lesion. Ischemic strokes caused by arterial vascular occlusion/stenosis account for 87% of arterial strokes.^1^ Cerebral venous sinus thrombosis as the main type of venous stroke is a particular subtype that is caused by interruption of venous blood flow due to thrombosis of the venous vessels, accounting for 0.5%–1% of strokes but accounting for 14%–20% of strokes in young adults.^2^ Importantly, the incidence of CVT has been rising with the advent of diagnostic techniques.

Compared to arterial stroke, CVT has the following characteristics: (1) low incidence rate, currently estimated at 13.2 to approximately 15.7 per million per year^3^, ^4^; (2) high prevalence in youth, female dominance, male to female ratio up to 1:3.5^5^; (3) the clinical manifestations are complex and varied, with great individual differences; (4) early diagnosis is difficult and often delayed or even missed, with a median delay of 7 days and a 73% missed diagnosis rate^6^, ^7^, ^8^; (5) risk factors for the disease are different from those for arterial stroke; (6) the drug regimens for the two stroke subtypes differ, with anticoagulation being the first‐line treatment option for CVT. These differences lead us to speculate that there may be fundamental differences in the molecular pathology of arterial and venous stroke. So far, extensive, and comprehensive mechanisms research has been performed on arterial stroke, but there are few studies on venous stroke, which limits the recognition of venous stroke and the development of related drugs.

Multi‐omics technologies offer the possibility of a more accurate elucidation of diseases by integrating the many interconnected and interacting components of biological systems to study the mechanisms of complex biological processes. Among them, transcriptome + metabolome research protocols have become popular in recent years, which can help us filter out key genes, metabolites, and metabolic pathways from a huge amount of data. In the study of intestinal inflammation, Liu et al. characterized the intestinal toxicity of BPF exposure by GC–MS untargeted metabolomic and transcriptomic study methods and explored the possible pathogenesis.^9^ By combining targeted metabolomic and transcriptomic studies of brain tissue samples to identify abnormalities in multiple metabolic networks associated with transmethylation and polyamine pathways in Alzheimer's disease, the study significantly increases the overall understanding of the metabolic basis of AD pathogenesis and provides insights into new targets for disease‐modifying therapies.^10^

To investigate the characteristic pathological mechanisms of venous stroke and arterial stroke, we compared the pathological mechanisms of the two‐stroke subtypes by transcriptomic and metabolomic techniques using an MCAO rat model to simulate arterial stroke and a CVT rat model to model venous stroke. Further, we explored the specific associations between transcriptomics and metabolomics through association analysis using bioinformatics technology, which contributes to a comprehensive understanding of the heterogeneity of arterial and venous strokes.

## 2 MATERIALS AND METHODS

### 2.1 Animals

In the present study, adult Sprague Dawley (SD, aged 8–10 weeks, each weighting approximately 250–300 g) rats were purchased from Vital River (Beijing, China). Each rat is housed in a conventional environment. Twelve‐hour light/dark cycle with free access to water and food for 1 week in the animal experiment center of Capital Medical University. The weight of each animal was controlled (300–350 g) during experiments. All animal experimental protocols were approved by the Institutional Animal Care and Use Committee of Beijing Capital Medical University.

### 2.2 The permanent MCAO rat model

MCAO surgery was performed according to the reference,^11^ Rats were deeply anesthetized with 4% of isoflurane and subsequently maintained between 1.5% and 2% during the surgical procedure using an isoflurane vaporizer (RWD, Shenzhen, China). Under anesthesia, the rats were placed in a supine position, using iodophor as a preoperative skin disinfectant, and a midline incision was made across the sagittal plane on the anterior neck, separating down the gland until the sternocleidomastoid was visible. Separating down the sternocleidomastoid, common carotid artery, external carotid artery, and internal carotid artery was exposed, isolating common carotid artery, external carotid artery, and internal carotid artery and their branches. The branch of the artery was ligated and cut. Artery clips were placed on the common carotid and internal carotid arteries, and the external carotid artery was ligated and cut to create a stump. A filament was inserted into the external carotid artery stump and then advanced into the middle cerebral artery which after removal of the artery clipped on the internal carotid artery until resistance was felt. Filaments were fixed at the external carotid artery stump and removal of the artery clip on the common carotid artery. The incision was sutured and sterilized again, and rats were housed in a separate cage after surgery.

### 2.3 The CVT rat model

The CVT rat model was performed as the previous method.^12^ Under anesthesia, the rats were placed in a prone position, using iodophor as preoperative skin disinfectant, and an incision was made in the middle of each rat's head, and the subcutaneous tissue was separated to expose the skull. A longitudinal cranial window, which exposed the SSS and bilateral cortex, using a high‐speed dental drill through microscopic observation. During the drilling process, the drill tip was continuously cooled with normal saline to avoid thermal injury in the dura mater and cortex. after exposing the SSS and bilateral cortex, semi‐ligated the SSS rostrally and caudally using an 8‐0 polyamide suture, and then thrombin was injected into SSS (200 μL/3 min). The surgical site was irrigated with normal saline, followed by sealing of the incision and sterilizing again. Once the surgery was completed, the wounds were closed using a 6‐0 surgical suture with a simple continuous pattern. Rats were allowed to recover under the heat lamp, and housed in a separate cage and antibiotic ointment was applied over the skin incision, following 5 consecutive days. All efforts were made to minimize the number of animals used and their suffering. In the sham group, the animals received only skull fenestration.

### 2.4 Sample preparation

The rats were randomly divided into three groups with nine rats in each: sham group, MCAO group, and CVT group. 48 hours after post‐surgery, rats in all groups were anesthetized with 1% pentobarbital sodium (40 mg/kg, intraperitoneal), then perfused with phosphate buffer saline. The brain was then removed from rats in the sham (*n* = 3), MCAO (*n* = 3), and CVT (*n* = 3) groups. After the material was taken, it was rinsed with 0.9% saline to remove blood and dirt, and the tissue types such as connective tissue and adipose tissue were removed and the liquid on the surface of the material was aspirated. Tissue samples were divided into small pieces of approximately 50 mg, quick‐freeze in liquid nitrogen, and stored in sterile storage tubes at −80°C until transcriptome sequencing and metabolomics analysis were performed.

### 2.5 RNA sequencing

Rat brain tissue was processed for total RNA extraction using PicoPure RNA Isolation Kit (# KIT0202 Arcturus, CA, USA) in accordance with the manufacturer's instructions, and genomic DNA was eliminated using a DNase treatment step in the kit. The amount and quality of the extracted total RNA were then examined using Agilent 2100 Bioanalyzer (NYSE: A, Palo Alto, USA) to verify the RNA samples. To create the sequencing library, only high‐quality RNA samples (OD260/280 = 1.8–2.2, OD260/230 = 2.0, RIN = 6.5, 28S:18S = 1.0, >2 mg) were employed. RNA‐seq transcriptome library was prepared following ABI StepOnePlus Real‐Time PCR System (Thermo Fisher Scientific, MA, USA) using 1 mg of total RNA. According to the principle of A‐T base pairing between magnetic beads with oligo (dT) and the polyA at the 3′ ends of mRNA, mRNA can be separated from total RNA for transcriptomic analysis. At the same time, we introduced a fragmentation buffer to break up big mRNA fragments into 300 bp fragments at random. Then the mRNA was reverse‐transcribed into cDNA, and after being connected to the adaptor, the mRNA was purified and amplified by PCR to obtain the final library. Finally, the paired‐end RNA‐seq sequencing library was sequenced with the DNBSEQ (SE50).

### 2.6 Read mapping

The raw data contains reads of low quality, reads with adaptor sequences, and reads with high levels of N base. Those reads were filtered before the data analysis for reliability analysis results. Then we use HISAT to align the clean reads to the reference genome. The Rattus_norvegicus genome (version of the reference genome: GCF_000001895.5_Rnor_6.0) was chosen as the reference genome, and the total mapping genome ratio ranged from 94.89% to 95.24%, the ratio of the uniquely mapping genome ranged from 83.99% to 85.92%.

### 2.7 Identification of differentially expressed genes

Using the transcripts per million mapped reads (TPM) method, we determined the expression level of each transcript to determine which genes were expressed differently in each sample. R statistical package software DEseq2 (http://www. bioconductor.org/packages/stats/bioc/edgeR/, version 3.14.0) was utilized for differential expression analysis. In addition, GO annotation analysis and KEGG function enrichment analysis (*p*‐adjust ≤0.05) are performed by Clusterprofiler (version 4.6.0).

### 2.8 Inflammation response analysis

The mouse MSigDB gene set (HALLMARK_INFLAMMATORY_RESPONSE) was used for the ssGSEA analysis. The inflammatory response score of each sample was quantified and completed using the R packages “GSVA” and “GSEABase.” 36 immune cell type fractions were calculated using ImmuCellAI‐mouse (http://bioinfo.life.hust.edu.cn/ImmuCellAI‐mouse/) in each sample which applied a hierarchical strategy to classify the 36 cell types into three layers.

### 2.9 Untargeted metabolomics analysis

A total of 50 mg of each sample was accurately weighed and transferred to a 2 mL centrifuge tube containing one small steel ball. Then 400 mL of the extraction (solvent methanol/water: 4/1, v/v) and 20 mL of the internal standard (2‐chloro‐Lphenylalanine) at a concentration of 0.02 mg/mL were added to the centrifuge tube. After 6 min of grinding in a frozen tissue grinder (−10°C, 50 Hz) and 30 min of low‐temperature ultrasonic extraction (5°C, 40 KHz), the sample was allowed to stand at −20°C for 30 min. Finally, the sample was centrifuged for 15 min, and the supernatant was transferred to a sample vial for LC–MS detection.

This project uses LC–MS/MS technology for untargeted metabolomics analysis, using high‐resolution mass spectrometer Q Exactive (Thermo Fisher Scientific, USA) to collect data from both positive and negative ions to improve metabolite coverage. LC–MS/MS data processing was performed using The Compound Discoverer 3.1 (Thermo Fisher Scientific, USA) software, which mainly included peak extraction, peak alignment, and compound identification. Data pre‐processing, statistical analysis, metabolite classification annotations, and functional annotations were performed using the was performed using MetaboAnalyst.^13^ The multivariate raw data is dimensionally reduced by PCA (Principal Component Analysis) to analyze the groupings in the data set (whether there is an abnormal sample).

### 2.10 Statistical analysis

Common tests for normality including the Shapiro–Wilk test were conducted. These tests evaluate the differences between the data sample and a normal distribution to determine if the data follow a normal distribution. If the data fail the normality test, non‐parametric equivalents such as the Wilcoxon rank‐sum test or Mann–Whitney U test were used for analysis. The significance was *p* < 0.05. ANOVA (analysis of variance), also known as “variance analysis,” is used to test the significance of differences among CVT, MCAO, and Sham. Metabolites that differed significantly (adjusted *p* < 0.05) were identified as differential metabolites.

## 3 RESULTS

### 3.1 Identification of differentially expressed genes

**FIGURE 1:**
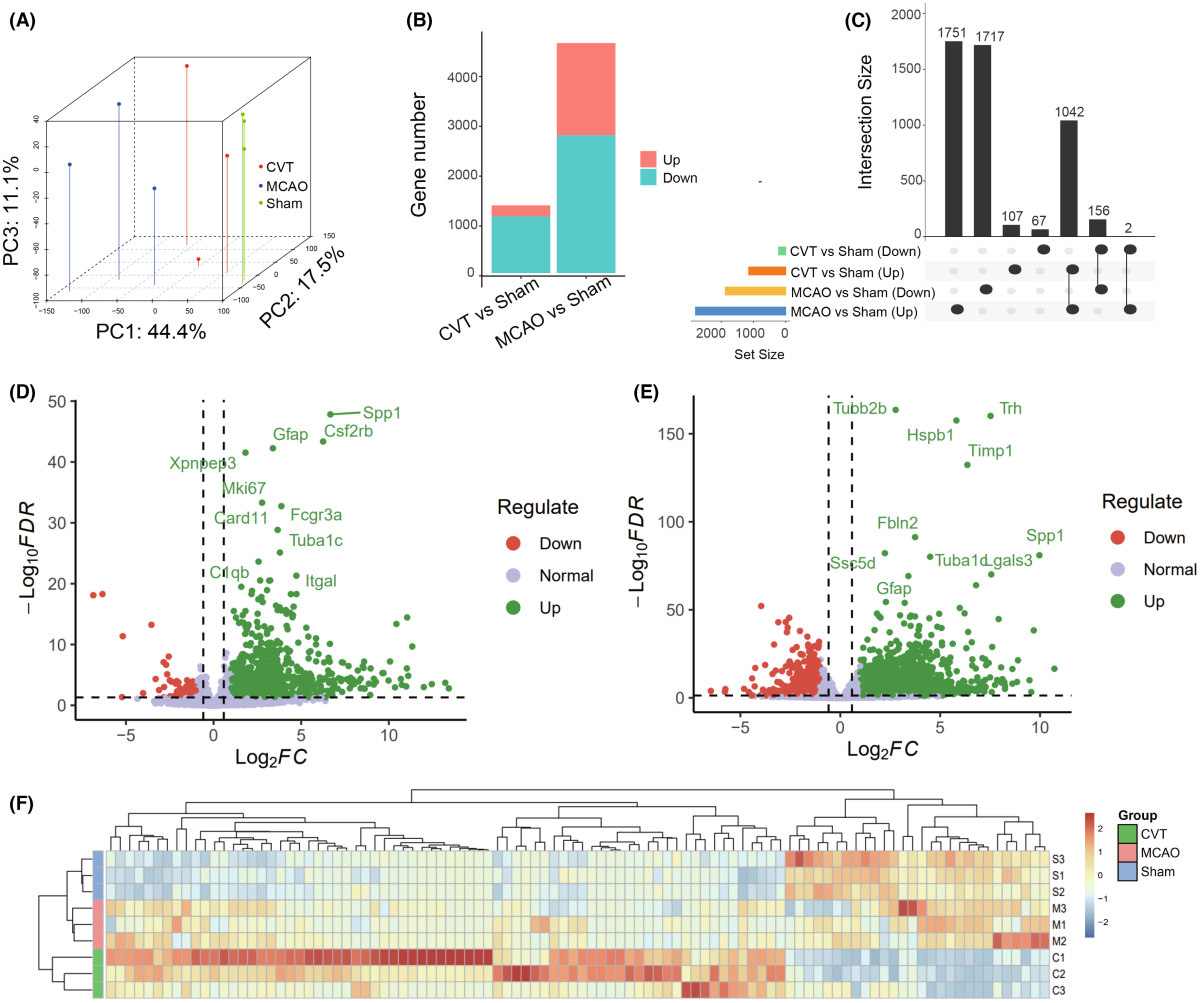
(A) Plots of Principal component analysis (PCA) of gene expression profiles of the three groups of CVT group, Sham group, and MCAO group. (B) The number of differentially expressed genes (DEGs, FDR adjusted *p* < 0.05) in the CVT group versus Sham group and MCAO group versus Sham. (C) Upset chart shows the intersections of the up and down DEGs between the two comparisons of the CVT group versus Sham group and MCAO group versus Sham genes. (D, E) The volcano plot shows DEGs of the CVT group versus the Sham group (D) and MCAO group versus Sham (E). Green dots indicate the upregulated genes and red ones are downregulated genes. (F) Heatmap of the CVT‐specific DEGs expression TPMs in three groups. Red rectangles represent the upregulated genes and blue ones are downregulated genes. The color depth means the significance of the difference.

Transcriptomic analysis was performed on the brain of rats, and we sequence nine samples using DNBSEQ SE50 platform, averagely generating about 1.19Gb bases per sample. The average mapping ratio with reference genome is 95.07%, and the average mapping ratio with gene is 70.29%; 19,980 genes were identified. After data quality control, a total of 214,526,500 clean reads and 10,726,325,000 clean bases were obtained. The proportion of each sample with quality scores ≥Q20 and ≥Q30 exceeded 97% and 93%, thus indicating the high quality of reads (Table S1). Principal component analysis (PCA) of gene expression profiles was performed, and the result showed good separations between Sham, MCAO, and CVT groups, indicating high correlation within the group and great differences between the Sham, MCAO, and CVT groups (Figure 1A). A total of 1372 different expressed genes (DEGs, FDR adjusted *p*‐value <0.05, fold change >1.5 or <0.67), including 1149 upregulated genes and 223 downregulated genes, were obtained in CVT versus Sham, and 4666 DEGs, including 2793 upregulated genes and 1873 downregulated genes, were obtained in MCAO versus Sham, respectively (Figure 1B, Table S2). The interaction result showed that the number of upregulated genes of CVT versus Sham and MCAO versus Sham was 1042, and the number of downregulated genes was 156. In total, 174 CVT‐specific DEGs were identified in the CVT group, of which 107 genes were only upregulated and 67 genes were only downregulated in CVT versus Sham, but not in CVT versus Sham (Figure 1C,D). Among *Spp1*, *Csf2rb*, *Gfap*, *Xpnpep3*, and *Mki67* were upregulated in CVT versus Sham significantly, importantly those genes have been reported to be associated with immune cell activation, proliferation, and glial cell activation (Figure 1D). *Tubb2b*, *Hspb1*, *Trh*, *Timp1*, and *Fbln2* were upregulated DEGs of MCAO versus Sham, and those genes have been reported to be associated with extracellular mechanism degradation and cell apoptosis (Figure 1E). The expression values of 100 CVT‐specific DEGs are shown in Figure 1F,

### 3.2 Pathway and function enrichment of DEGs

**FIGURE 2:**
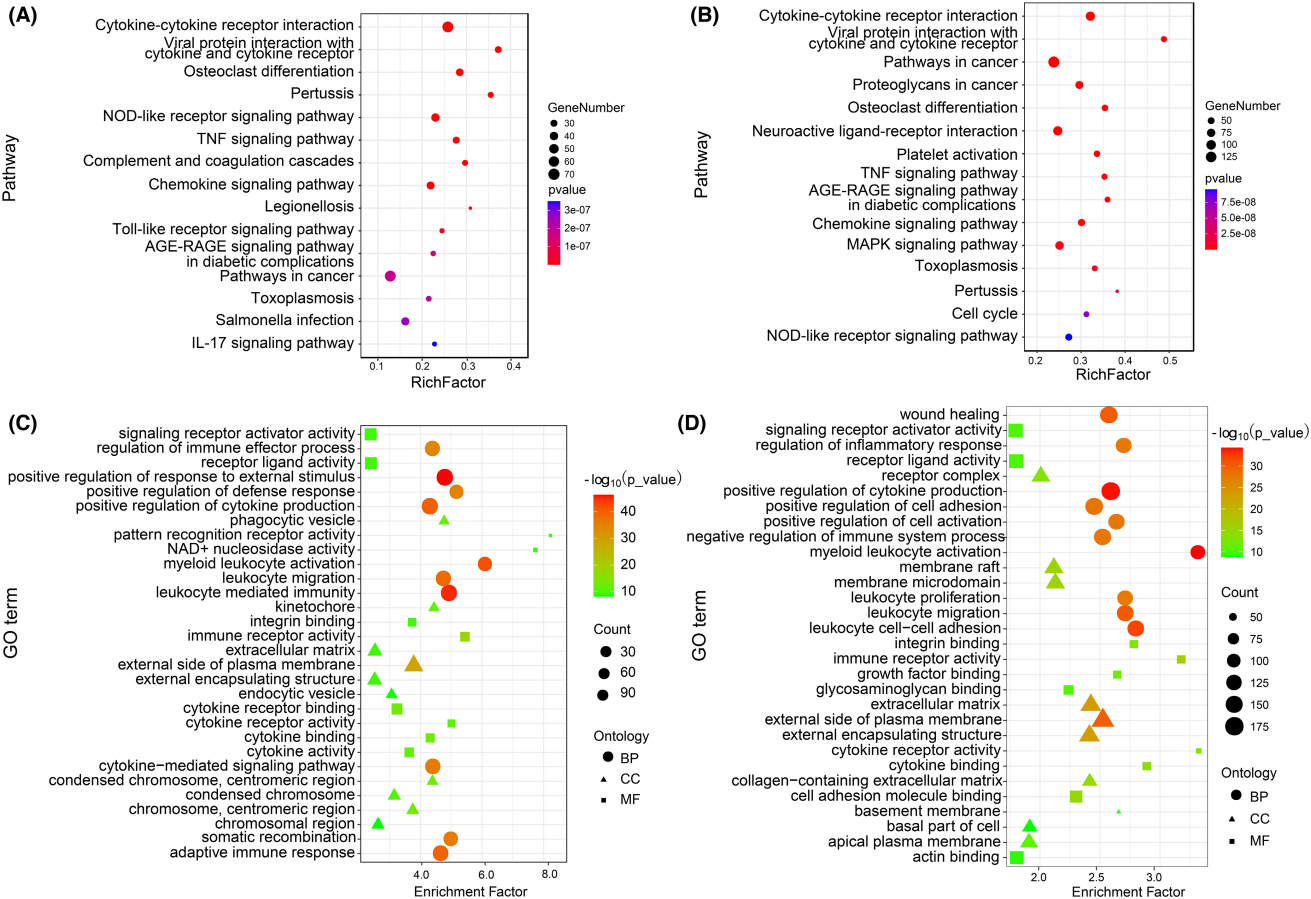
The top 15 enriched KEGG pathways of DEGs in the CVT group versus Sham group (A) and MCAO group versus Sham (B). The color means the adjusted *p*‐value and the area of bubbles shows the number of DEGs enriched. Gene Ontology (GO) enrichment analysis of DEG in CVT group versus Sham group (C) and MCAO group versus Sham (D) of the three GO categories. The dot color means the adjusted *p*‐value and the area of bubbles shows the number of DEGs enriched. The dot shapes represent the three GO term categories.

We performed a detailed analysis of the DEGs of the two groups, including Gene ontology (GO) and the KEGG pathway. In the CVT versus Sham group, the top 15 significantly enriched pathways were screened by KEGG enrichment results, including cytokine‐cytokine receptor interaction, viral protein interaction with cytokine and cytokine receptors and pathways in cancer (Figure 2A). The top 20 of GO enrichment showed that in the molecular function category, most DEGs were associated with binding, catalytic activity, and molecular transducer activity. In the cellular component category, the dominant subcategories were cell, cell part, and organelle. Among the biological processes, the cellular process, single‐organism process, and biological regulation were most enriched (Figure 2C). In the MCAO versus Sham group, the top 15 significantly enriched pathways were screened by KEGG enrichment results. Including cytokine‐cytokine receptor interaction, viral protein interaction with cytokine and cytokine receptors, and osteoclast differentiation (Figure 2B). And the top 20 of GO enrichment were consistent with the results of CVT versus Sham (Figure 2D).

### 3.3 CVT special genes and pathways

**FIGURE 3:**
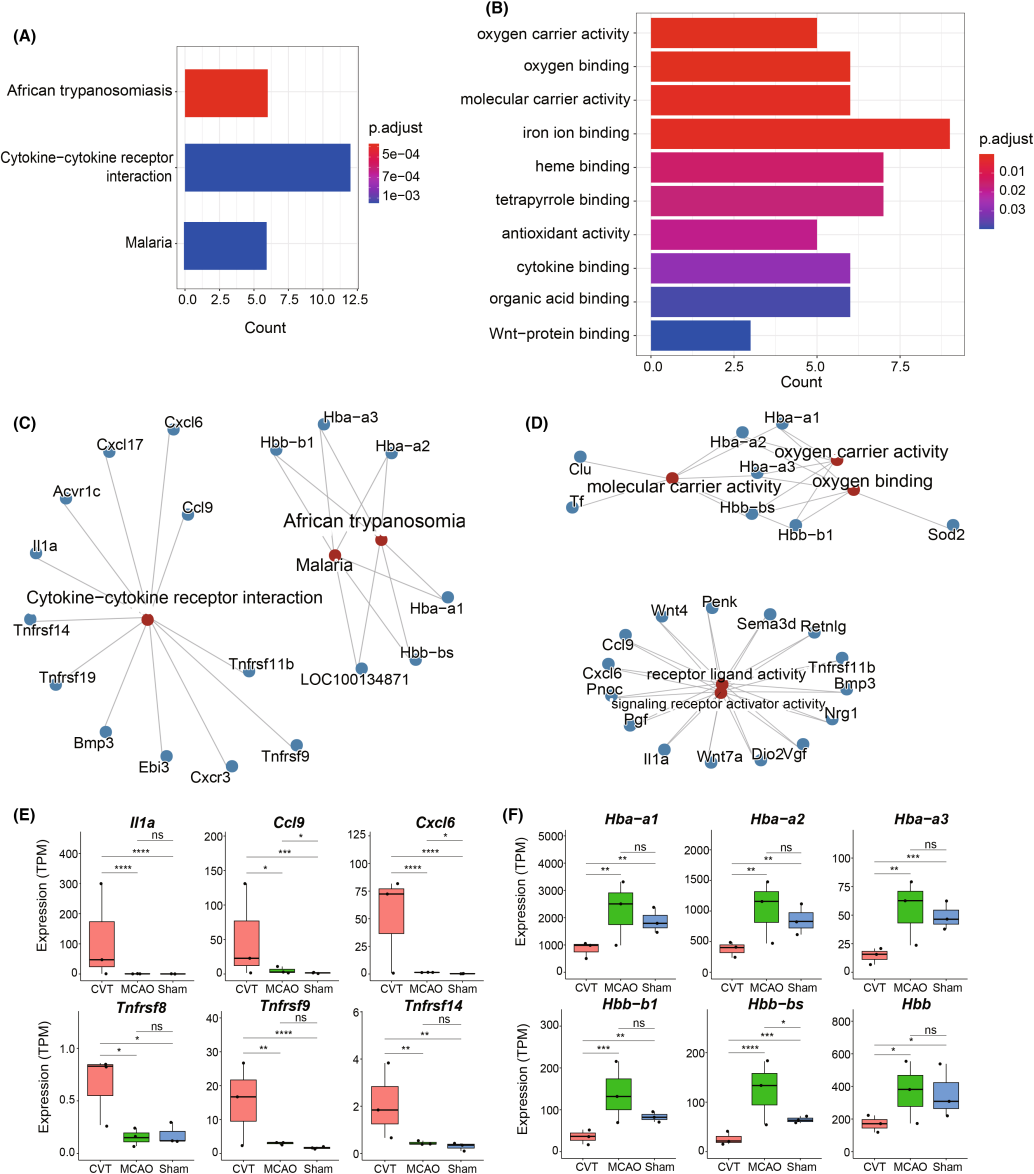
KEGG (A) and GO annotation (B) of CVT‐specific DEGs. Network plot of enriched KEGG (C) and GO (D) terms. (E, F) Mean value for genes enriched in Cytokine−cytokine receptor interaction pathway (E) and oxygen carrier activity (F) (DEseq2 adjusted *p*‐value, *p < 0.05; **p < 0.01, ***p < 0.001).

Enrichment of functions and pathways analysis were performed on 174 DEGs based on GO and the Kyoto Encyclopedia of Genes and Genomes (KEGG) database, the result showed that CVT‐specific genes were mainly enriched in African trypanosomiasis, cytokine‐cytokine receptor interaction and Malaria pathway(Figure 3A,C), and the MCAO group was mainly enriched in oxygen carrier activity, oxygen binding, molecular carrier activity and iron ion binding pathway (Figure 3B,D).

**FIGURE 4:**
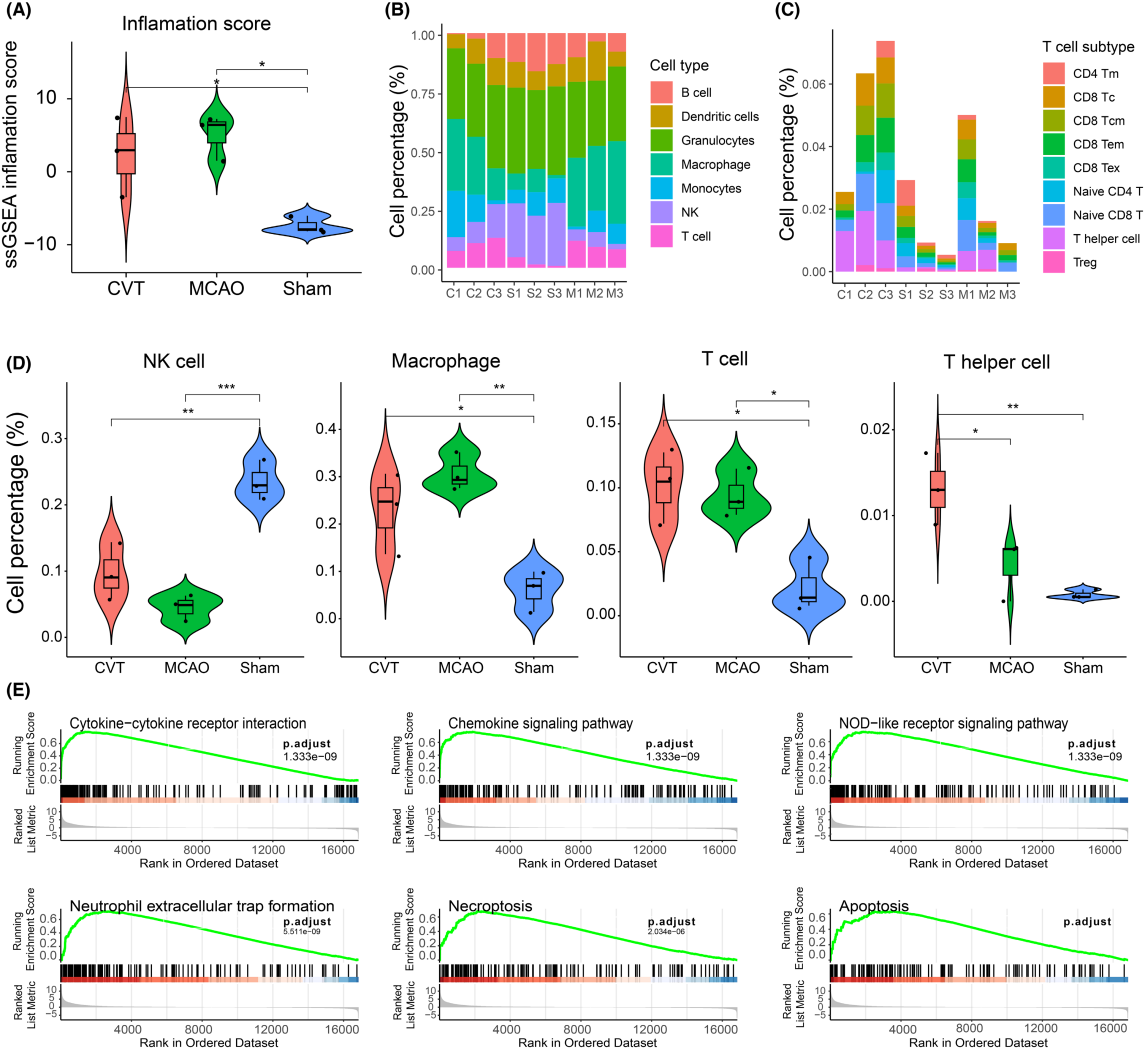
(A) Violin plot indicates the inflammation differences in three groups, showing the median, quartile, and kernel density estimations for the immune response score. (B, C) The relative proportion of seven major immune cells (B) and T‐cell subtypes (C) in each sample. (D) Mean value for each cell subset including, NK cells, Macrophages, T cells, and T helper cells, was calculated for each group and compared (one‐way ANOVA analysis, Tukey post hoc tests, *p < 0.05; **p < 0.01, ***p < 0.001). (E) GSEA analysis reveals the enriched KEGG pathways related to inflammation response in CVT.

Gene level analysis showed that the expression values of interleukin family genes *IL1a*, *Ccl9* and *Cxcl6* were significantly higher in the CVT (Figure 3E). IL1a is mainly involved in various immune responses, inflammatory processes, and hematopoiesis. On chromosome 2, this gene and eight other genes from the interleukin 1 family form a cytokine gene cluster. These genes' polymorphisms have been linked to rheumatoid arthritis and Alzheimer's disease, according to some research.^14^ These genes play important roles in innate and adaptive immunity and are involved in T cell‐related responses. It was also found that the expression values of *Tnfrsf8*, *Tnfrsf9*, *Tnfr*, and *sf14* of the tumor necrosis factor receptor superfamily had no significant difference between the MCAO and Sham groups while they tended to be higher in the CVT group. Except for Tnfrsf18, the expression values of Tnfrsf8, Tnfrsf9, and Tnfrsf14 genes in CVT group were significantly higher than those in the MCAO group (Figure 3E). This superfamily member is mainly related to T cell and B cell expression and participates in T cell and B cell‐related inflammatory immune response. On the contrary, expression values of genes related to the family of hemoglobin *Hba‐a1*, *Hba‐a2*, *Hba‐a3*, *Hbb‐b1*, *Hbb‐bs*, and *Hbb* were lower in the CVT group, and significantly lower than in the MCAO group (Figure 3E). The genes of this family are mainly involved innate immune system and O_2_/CO_2_ exchange in erythrocytes and cellular responses to stimuli. GO annotations related to these genes include iron ion binding and oxygen binding. Using the ssGSEA method, the hallmark inflammatory GSEV z‐score was quantified and it showed that both CVT and MCAO mice had a higher inflammatory response (Figure 4A). ImmuCellAI‐mouse (Immune Cell Abundance Identifier for mouse) was used to deconvolute the abundances of 36 types of cells, showing the differences in the CVT, MCAO, and Sham groups (Table S3). Figure 4B summarizes the abundance profiles of 7 major immune cells of the first layer derived from ImmuneAImouse. In detail, CVT and MCAO exhibited substantially higher levels of T cell and macrophages and decreased proportions of NK cells. The abundance of T cell subtypes including T helper cells was higher in CVT (Figure 4C,D). The abundance of the other types of cells is shown in (Figure S1A,B). GSEA analysis showed that CVT had a significant activation of the immune pathways, including cytokine−cytokine receptor interaction, chemokine signaling pathway, NOD‐like receptor signaling pathway, and neutrophil extracellular trap formation, necroptosis, and apoptosis (Figure 4E).

### 3.4 Metabolites analysis

**FIGURE 5:**
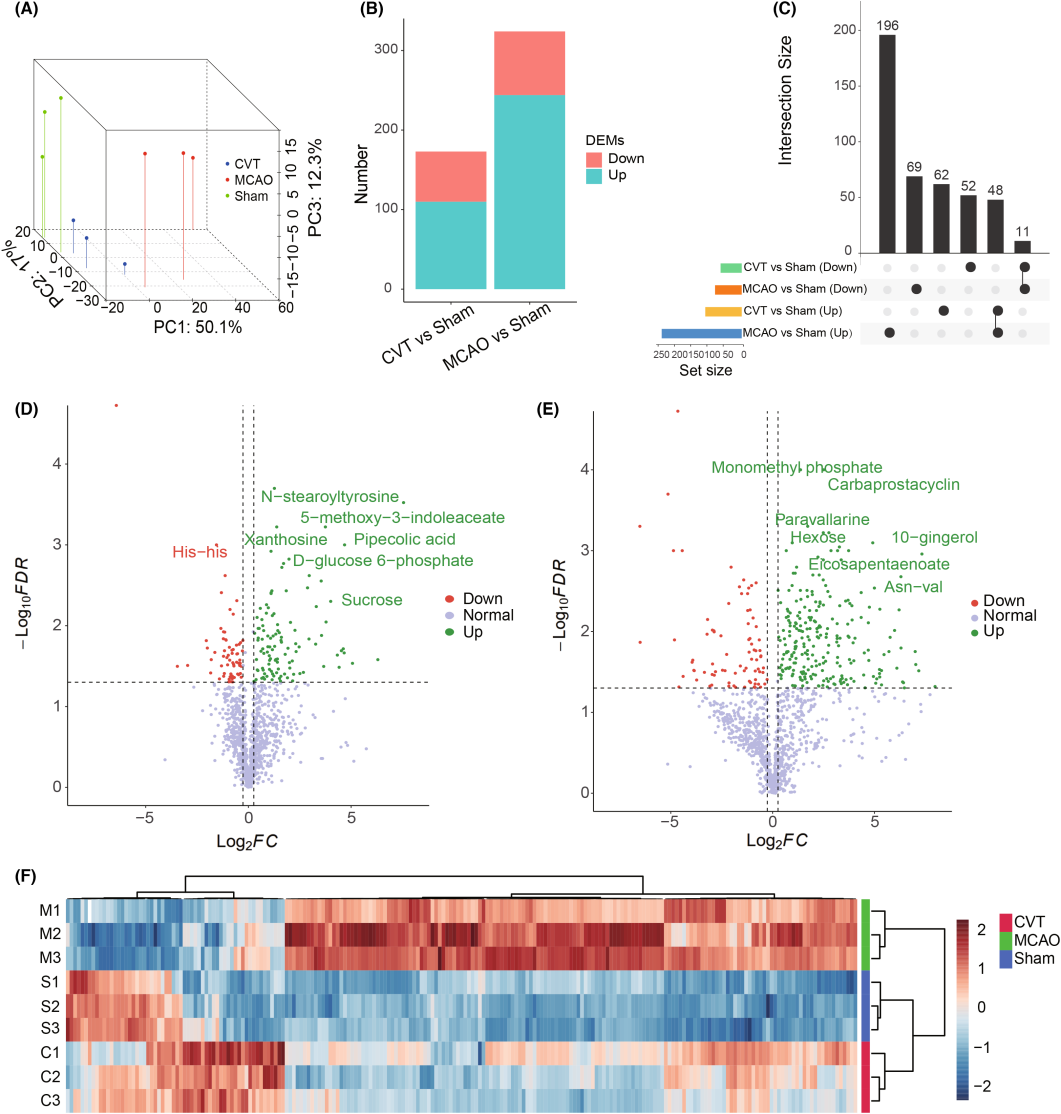
(A) Plots of Principal component analysis (PCA) of metabolism profiles of the three groups of CVT group, Sham group, and MCAO group. (B) The number of differential metabolites (DEMs, *p*‐value <0.05, Fold Change >1.2 or <0.83) in the CVT group versus the Sham group and MCAO group versus Sham. (C) UpSet chart shows the intersections of the up and down DEMs between the two comparisons of the CVT group versus Sham group and MCAO group versus Sham genes. (D, E) The volcano plot shows DEMs of the CVT group versus Sham group (D) and MCAO group versus Sham (E). Green dots indicate the upregulated metabolites and red ones are downregulated metabolites. (F) Heatmap of DEMs derived from one‐way ANOVA test of the three groups(one‐way ANOVA analysis, *p* < 0.05). Red rectangles represent the upregulated metabolites and blue ones are downregulated metabolites. The color depth means the significance of the difference.

**FIGURE 6:**
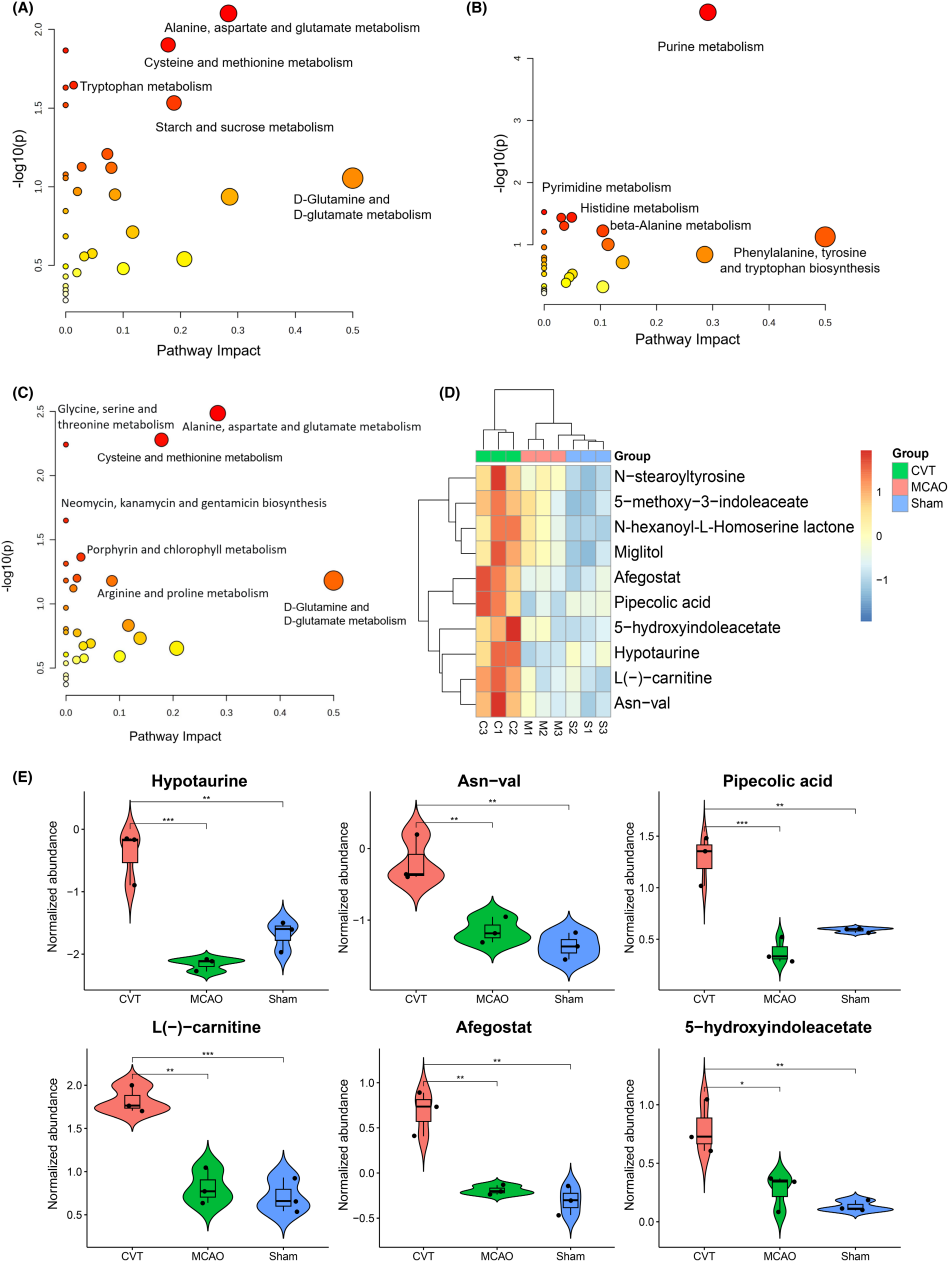
Pathway enrichment analysis of DEMs in CVT group versus Sham group (A) and MCAO group versus Sham (B) and CVT‐specific DEMs (C). (D) Heatmap of metabolites increased in CVT derived from ANOVA one‐way test of the three groups. Red rectangles represent the upregulated metabolites and blue ones are downregulated metabolites. The color depth means the significance of the difference. (E) Mean value for DEMs specific in CVT group versus Sham group (one‐way ANOVA analysis, Tukey post hoc tests, *p < 0.05; **p < 0.01, ***p < 0.001).

In this study, non‐targeted metabolomics was used to analyze the differential metabolites (Table S4). PCA analysis was also performed for metabolism (Figure 5A). The histogram shows the up‐ and downregulation of differential metabolites in CVT versus Sham and MCAO versus Sham (Figure 5B, Tables S5 and S6). The Venn showed that compared with the Sham group, the number of different metabolites in CVT groups was 110 significantly upregulated ones and 63 downregulated ones (Figure 5C,D). The number of different metabolites in MCAO groups was 244 significantly upregulated ones and 80 downregulated ones (Figure 5C,D). The volcano plot of metabolites shows the up‐down of differential metabolites in CVT versus Sham and MCAO versus Sham (Figure 5E,F). One‐way ANOVA test was also performed to select the different metabolites in three groups (Figure 5G, Table S7, one‐way ANOVA analysis, *p* < 0.05). To explore the potential metabolic pathways affected, we further analyzed all DMs based on KEGG annotations. The most enriched pathways in the CVT group compared with the Sham group were alanine, aspartate, and glutamate metabolism cysteine and methionine metabolism; tryptophan metabolism; Starch and sucrose metabolism; and d‐glutamine and d‐glutamate metabolism (Figure 6A). The most enriched pathways in the MCAO group were purine metabolism; pyrimidine metabolism; histidine metabolism; beta‐alanine metabolism; and phenylalanine, tyrosine, and tryptophan biosynthesis (Figure 6B). Based on one‐way ANOVA analysis, the metabolism levels of N‐stearoyltyrosine, 5‐methoxy‐3‐indoleaceate, 3‐hydroxy‐n‐[(3s)‐2‐oxotetrahydro‐3‐furanyl]octanamide miglitol, afegostat, pipecolic acid, 5‐hydroxyindoleacetate, hypotaurine, l(−)‐carnitine and Asn‐val, etc., are significantly increased in the CVT group, but not in MCAO(Figure 6D,E). Figure S2 shows the test results for these metabolites. It has been demonstrated that indole‐3‐acrylic acid reduces DNA damage and lipid peroxidation, protecting neurons from ischemia‐induced damage.^15^ Tacrine‐8‐hydroxyquinoline hybrid has the potential to treat AD‐related brain damage and may have a significant beneficial effect on neurodegenerative diseases.^16^ A class of compounds known as 8‐hydroxyquinoline has been found to have the potential to treat a number of neurodegenerative diseases.^17^, ^18^

## 4 DISCUSSION

Currently, it has been little known about the CVT mechanism, especially the immune response. Some studies reported that the inflammatory reaction may be an essential factor in the development of severe CVT and is strongly associated with poor prognosis.^19^, ^20^, ^21^, ^22^, ^23^, ^24^ This study found a more significant state of immune activation in brain tissue after venous stroke compared to arterial stroke models in rat models, which is consistent with previous studies.^25^

A better understanding of the mechanism after CVT is beneficial for early prevention and treatment. In the present study, we investigated the transcriptional profiles of rat brains after CVT and also MCAO and found the upregulated DEGs and downregulated DEGs. These CVT‐specific regulated DEGs were obviously enriched in inflammatory response, and oxygen carriers. In addition, cell enrichment analysis and immune infiltration analysis also found several inflammatory cells, such as NK cells, macrophages, and T cells, especially T helper cells, were more significantly infiltrated into the brain after CVT. Accordingly, our results demonstrated that several genes involved in the inflammatory response, including well‐known proinflammatory genes *Il1a*, *IL23*, *Cxcl6*, *Cxcl1*, and genes specially expressed in immune cells such as *Tnrsf9* and *Tnrsf11b* were higher in CVT, which will provide potential diagnosis and treatment targets for CVT. It was also found that there was a substantial upregulation of the immune pathways in CVT, including cytokine−cytokine receptor interaction, chemokine signaling pathway, NOD‐like receptor signaling pathway, neutrophil extracellular trap formation, necroptosis, and apoptosis. Recent study in mouse CVT models found that the levels of phosphorylated‐NF‐κb p65, ROS, and TXNIP are distinctly raised post‐CVT, which synergistically conduce to the activation of the NLRP3 inflammasome, and then played a proinflammatory role.^25^ The present study also found significant changes in the inflammatory response after venous stroke in a rat CVT model, complementing the understanding of the inflammatory response after CVT.

Studies have shown that hypoxia is a major post‐stroke crisis in stroke patients, and continuous provision of oxygen to improve the poor microenvironment may effectively protect neurons from ischemic brain damage.^26^ Wang et al. developed a nanophotosynthesis biosystem to generate oxygen and promote angiogenesis and brain tissue repair, thus saving ischemic neurons and achieving the purpose of stroke treatment.^27^ Early studies have shown that hyperbaric oxygen therapy can significantly improve neurological function in stroke patients.^28^ Hyperbaric oxygen therapy is used to treat various neurological disorders associated with hypoxia, such as AD, ischemia, and traumatic brain injury.^29^ The previous study suggests a connection between the neuroactive ligand–receptor interaction and α‐synuclein, which plays a role in the brain dysregulation of ion miRNAs.^30^ Neuroactive ligand–receptor interactions were found to be a potential target of Tao‐Hong‐Si‐Wu treating middle cerebral artery occlusion therapeutic target.^31^ Over 100 genes involved in neuroactive ligand–receptor interaction and cytokine–cytokine receptor interaction were found to undergo significant pathway changes in research on the mechanism of tauopathy in autism.^32^ Cytokine–cytokine receptor interaction plays a crucial role in immune and inflammatory responses to diseases. In the brain, cytokine action is also influenced by the interaction of cytokines with hormones, neurotransmitters, peptides/neuropeptides, and neurotransmitters. During health and disease, cytokines act as immunomodulators and neuromodulators in the central nervous system, and their direct effects on neuronal cells can increase or decrease neuronal activity.^33^ Under certain pathophysiological conditions, cytokine cascades can lead to neurotoxicity and neurodegeneration.^34^ The cytokine–cytokine receptor interaction pathway was found to be enriched in the study of major influence pathways in neurodegenerative diseases.^35^

In addition, our results revealed that hemoglobin proteins, including both *Hba* and *Hbb*, were significantly downregulated after CVT, compared both to MCAO and Sham groups. These results also suggest that the mechanisms of post‐CVT pathology may be involved in oxygen binding and carrier and system inflammation, and need to be explained in the future.

Changes in the metabolome have been linked to several neurodevelopmental and neurodegenerative disorders. The current study includes a comprehensive profiling of the metabolites from CVT, MCAO, and Sham samples. We observed that specific metabolites and metabolic pathways including alanine, aspartate, and glutamate metabolism and cysteine and methionine metabolism were altered in CVT, and suggest significant heterogeneity in venous stroke relative to arterial stroke in terms of metabolic characteristics.

In summary, the present study provided an extensive analysis of DEGs and DEMs and revealed a series of targets and pathways involved in the inflammatory response and oxygen binding and carrying, and metabolism. These findings add significant insights into the pathogenesis mechanism of CVT and expand our understanding of the heterogeneity of venous stroke.

## AUTHOR CONTRIBUTIONS

Ling Kui, Chen Zhou, and Yinming Jiao participated in the experimental design, analyzed sequencing datasets, and wrote the paper. Chen Zhou, Zongyu Li, Huimin Jiang, and Guoyun Wang conducted animal and performed experiments. Ling Kui, Yinming Jiao, and Zongyu Li conducted the bioinformatics analyses. Xunming Ji supervised this project and revised the manuscript.

## FUNDING INFORMATION

This work was supported by the Cheung Kong (Changjiang) Scholars Program (T2014251) and the National Natural Science Foundation of China (82271311).

## CONFLICT OF INTEREST STATEMENT

The authors have stated that they had no interests that might be perceived as posing a conflict or bias.

## Supporting information


Figure S1A.

Figure S1B.

Figure S2.

Table S1.

Table S2.

Table S3.

Table S4.

Table S5.

Table S6.

Table S7.

## Data Availability

Data available on request from the authors.
