# Supplementary figures and images for: Expression and metabolism profiles of CVT associated with inflammatory responses and oxygen carrier ability in the brain
Source: CNS Neurosci Ther. 2023 Oct 30;30(4):e14494. doi: 10.1111/cns.14494 (PMC11017414; doi:10.1111/cns.14494)

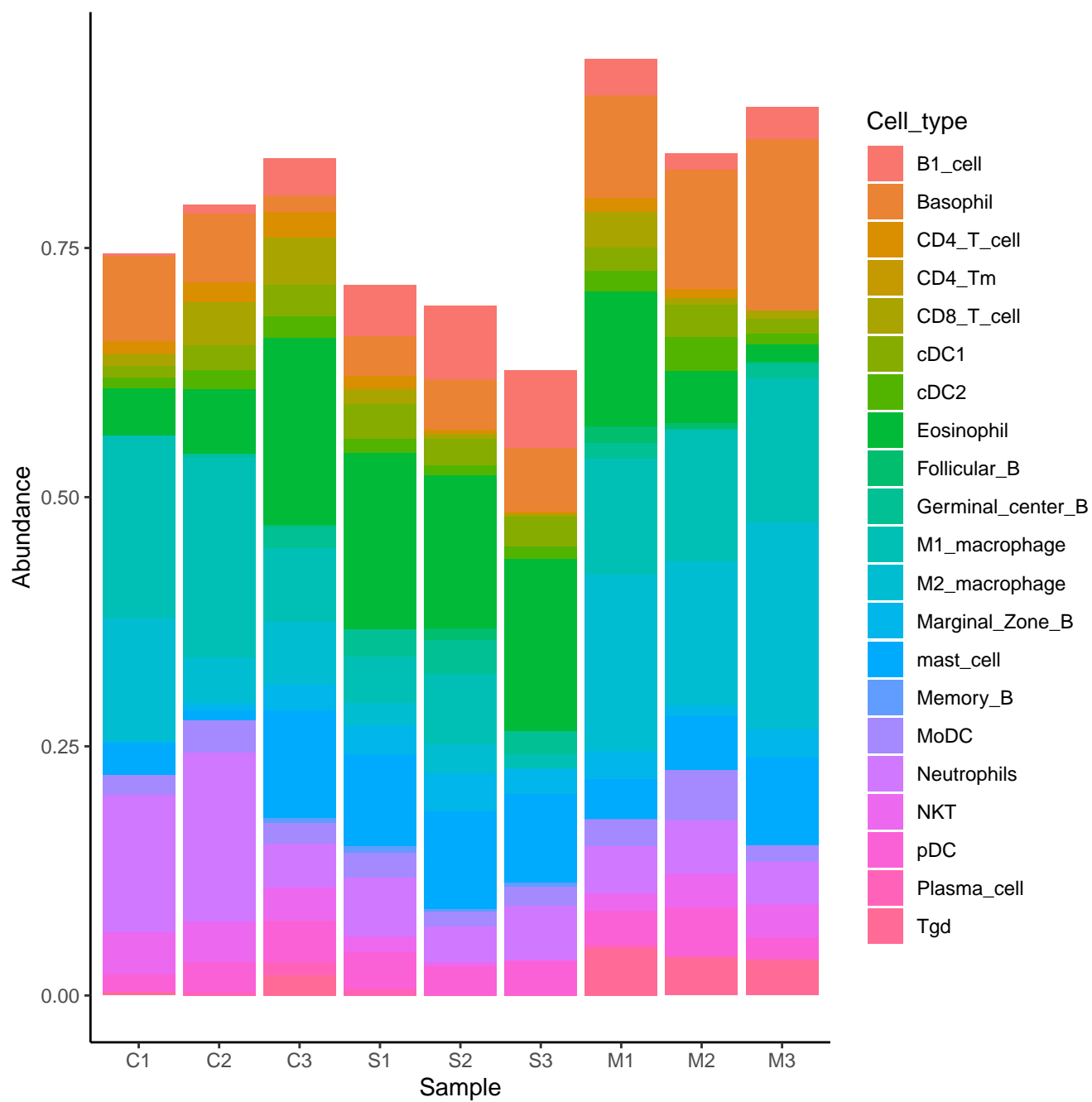

Supplement: Supplementary file 1 — Figure S1A. [file CNS-30-e14494-s006.pdf]

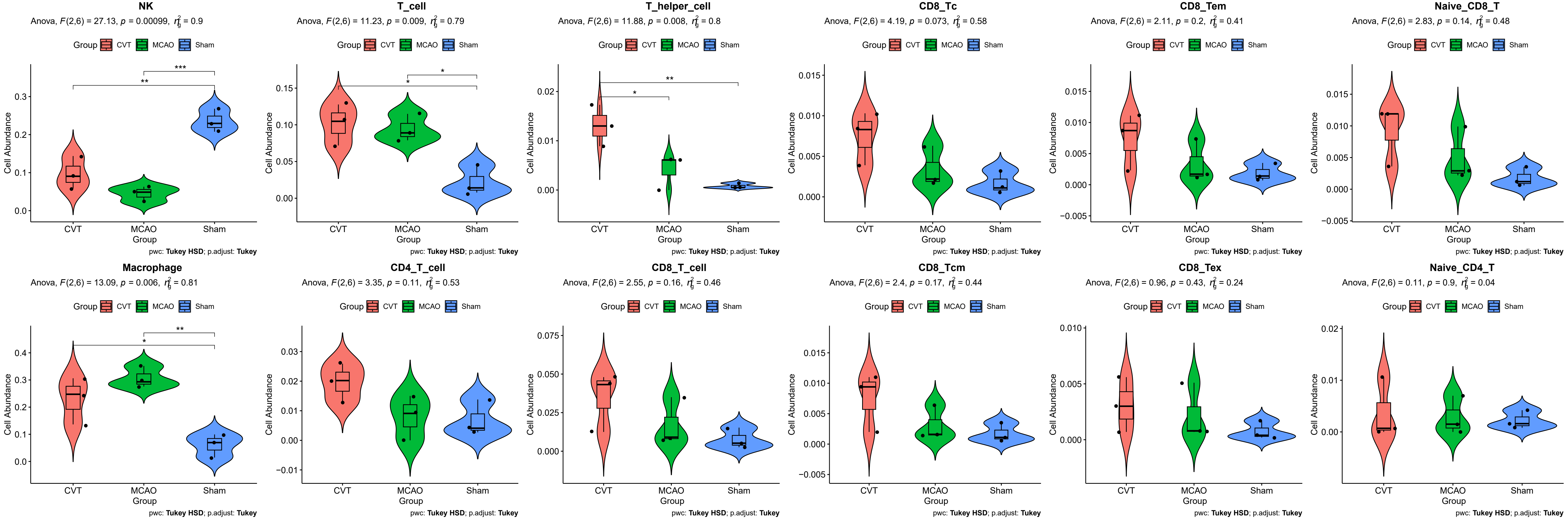

Supplement: Supplementary file 2 — Figure S1B. [file CNS-30-e14494-s002.pdf]

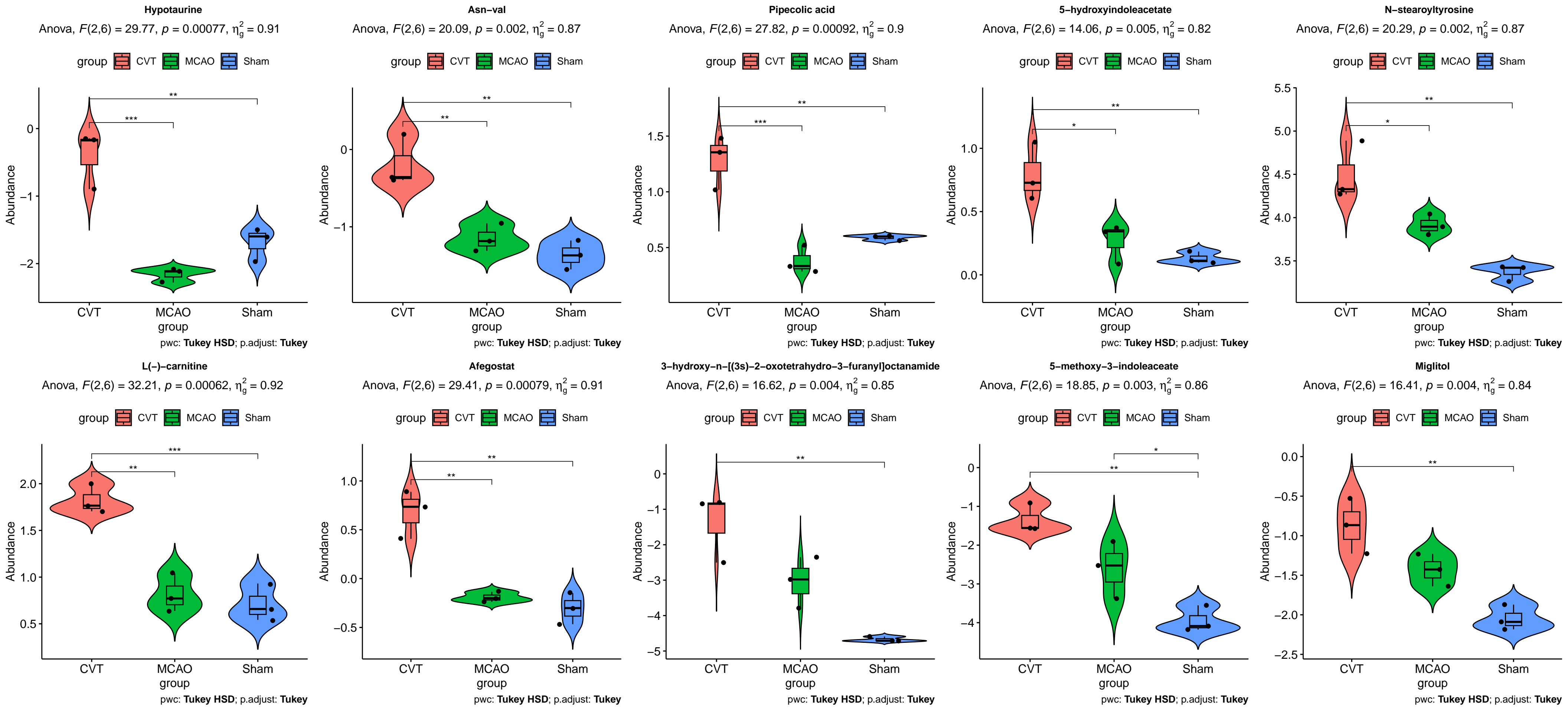

Supplement: Supplementary file 3 — Figure S2. [file CNS-30-e14494-s008.pdf]
